# Coronary Artery Surgery: Past, Present, and Future

**DOI:** 10.5041/RMMJ.10515

**Published:** 2024-01-19

**Authors:** Elizabeth C. Ghandakly, Gabriele M. Iacona, Faisal G. Bakaeen

**Affiliations:** Coronary Center, Department of Thoracic and Cardiovascular Surgery, Heart, Vascular & Thoracic Institute, Cleveland Clinic, Cleveland, Ohio, USA

**Keywords:** CABG, conduits, internal thoracic artery, multiple arterial grafting

## Abstract

Coronary artery bypass grafting (CABG) is the most commonly performed and studied major cardiac operation worldwide. An understanding of the evolution of CABG, including the early days of cardiac surgery, the first bypass operation, continuous improvements in techniques, and streamlining of the operation, is important to inform current trends and future innovations. This article will examine how CABG evolved (from techniques to conduits), describe current trends in the field, and explore what lies on the horizon for the future of CABG.

## INTRODUCTION

Coronary artery bypass grafting (CABG) is the most commonly performed and studied major cardiac operation, both in the United States and worldwide, with almost 400,000 procedures performed each year in the United States alone.^1^,^2^ This review ex-amines the evolution of coronary revascularization that has led to modern-day CABG practice. This includes a brief historical review of coronary revascularization, a discussion of major trials that led to current guidelines, an examination of present-day trends in CABG, and a look at what future innovations and trends lie on the horizon.

## CABG: A BRIEF HISTORY

### Vascular Discovery and Palliative Care of Chest Pain: 1900–1946

**Figure 1:**
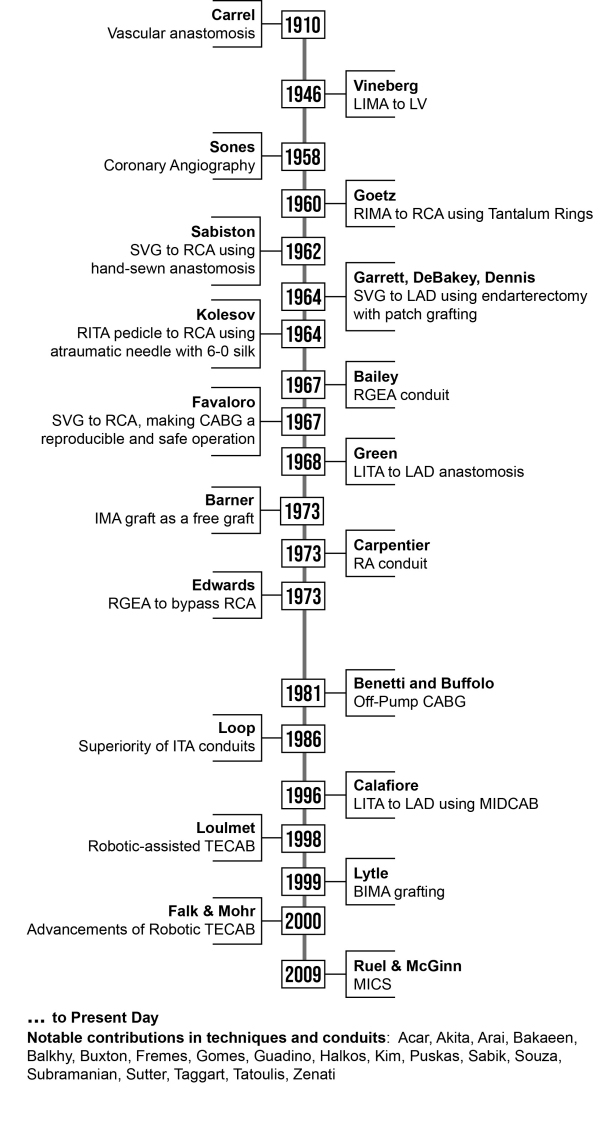
Timeline of Important Contributions in CABG BIMA, bilateral internal mammary artery; CABG, coronary artery bypass graft; IMA, internal mammary artery; ITA, internal thoracic artery; LAD, left anterior descending coronary artery; LIMA, left internal mammary artery; LITA, left internal thoracic artery; LV, left ventricle; MIDCAB, minimally invasive direct coronary artery bypass; MICS, minimally invasive coronary artery bypass; RA, right atrium; RCA, right coronary artery; RGEA, right gastroepiploic artery; RIMA, right internal mammary artery; RITA, right internal thoracic artery; SVG, saphenous vein graft; TECAB, totally endoscopic coronary artery bypass.

The concept of operating on the coronary vascula-ture was first described by the Nobel Laureate, vascular surgeon Alex Carrel in 1910. Carrel, in a time before the development of polypropylene su-tures and atraumatic needles, had a fascination with the possibility of performing vascular anastomoses (Figure 1). Driven by this fascination, and the need to treat wartime wounds, he experimented prolif-ically on dogs. This led to the development of not only a rudimentary device to keep organs alive out-side the body for transplantation and his more well-known Carrel–Dakin method for treating war wounds, but also successful intrathoracic aortic and coronary anastomoses in dogs.^3^

Nevertheless, surgeons long believed it to be a fool’s errand to attempt surgery on the human heart. As a result, all efforts at the beginning of the last century were directed toward palliation of chest pain. In 1930, Claude Beck noticed that scars and adhesions that were induced in the pericardium were highly vascular. Thus, he sought to increase myocardial blood supply by inducing new vascular anastomoses between coronary arteries and sur-rounding tissues (such as pericardial fat, omentum, and muscle pedicles placed inside the pericardium after poudrage). Beck used this technique to treat angina pectoris in humans and attempted the first cardiomyopexy in 1935.^4^

### Indirect Myocardial Revascularization: 1946–1956

An innovative approach to the treatment of myocar-dial ischemia was derived from the experiments of Arthur Vineberg, with the creation of his “Vineberg Operation.” The conception of this procedure arose from a discussion Vineberg had with colleague Eric MacNaughten after a strenuous cardiovascular gym workout following a lecture regarding the pathology of coronary artery disease (CAD).^5^ He knew of bridging myocardial sinusoids from earlier work by Wearn in 1933, and of Beck’s cardiomyopexy work already described herein. Drawing on the concept that the pathology involved surface coronary arteries and their epicardial branches, Vineberg proposed that another artery’s branches could join intramyo-cardial arterioles, and that the left internal thoracic artery (LITA) would be best suited to the task. In 1946, he implanted the LITA directly into the left ventricle to relieve myocardial ischemia.^3^ It was not until 1951, however, that Vineberg reported this success.

This procedure became popular due to its acceptable mortality rate mixed with its relative success in relieving angina. In Vineberg’s procedure, collateral vessels mature because of bleeding of the internal thoracic vessels into the surrounding tissue. It was later proven successful through angiography, as described in the text that follows, and validated by a large Cleveland Clinic study of over 1,100 pa-tients.^5^ In fact, 54% of patients studied with post-procedure coronary angiography exhibited collateral vessels at the implant site of the LITA. Of note, this procedure inspired the LITA to left anterior de-scending coronary artery (LAD) operation that is done today.^6^ In 1956, Charles Bailey approached the same problem of angina by performing coronary endarterectomies.^3^

### Direct Myocardial Revascularization: 1958–1968

The years between 1958 and 1968 were some of the most exciting and dynamic in the history of cardiac surgery (Figure 1). Following World War II, enor-mous financial resources from the US government were invested in research. Scientists, surgeons, and private companies joined forces, leading to impor-tant innovations including the discovery of heparin and invention of the cardiopulmonary bypass ma-chine, blood oxygenator, and cardioplegic solutions.^7^ As a natural progression, many physicians from the Western world were attracted by the challenge of relieving cardiac symptoms in patients with myocar-dial ischemia.

Even as these innovations targeting coronary circulation to treat angina evolved, there was no method by which physicians could directly visualize the coronary circulation to determine where prob-lems existed. A paradigm shift that allowed such direct visualization occurred by happenstance—as so many breakthroughs do—in 1958 during Mason Sones’s treatment of a young patient with rheumatic heart disease at the Cleveland Clinic, in Cleveland, OH, United States.^8^ While completing an aortogram in this patient with mitral disease, Sones’s catheter engaged the ostium of the right coronary artery (RCA). In the process of mobilization in attempting to remove it, a puff of dye was accidentally injected into the RCA. The patient survived, and Sones visu-alized the flow through the artery, leading him to conclude that injection into the coronary arteries was feasible.^9^ Building on this event, Sones inten-tionally injected contrast dye into subsequent pa-tients and thus invented modern-day coronary angi-ography. He also thereby proved the merits of the Vineberg procedure by demonstrating, angiogra-phically, the connection between the implanted LITA and the myocardial vessels.

Of note, Cleveland Clinic had already been engaged in devising a method to visualize coronary arteries, but catheter introduction into the ostia was deemed too bold and risky. Sones’s discovery effectively transformed myocardial revascularization from a laboratory to a clinical procedure.^10^

The fear of coronary surgery was slowly vanish-ing. In 1959, Charles Dubost, of France, became the first surgeon to perform a coronary artery operation in a human using cardiopulmonary bypass when he performed an endarterectomy on a patient with syphilitic aortitis. This success with cardiopulmonary bypass would pave the way to allow for more com-plex surgeries to come.^11^ Around the same time, in 1961 in Sweden, Ake Senning was able to use a pericardial patch to enlarge the left main coronary artery, and, just months later at the Cleveland Clinic, Donald Effler used this pericardial patch technique by applying it to both left and right coro-nary arteries.^3^

In 1960, at the Albert Einstein College of Medi-cine, in New York City, NY, US, Robert Goetz per-formed the first successful CABG in a human using Rosenak (tantalum) rings. Famously, after having developed these surgical skills by performing suc-cessful bypass procedures on dogs, Goetz and his team were able to anastomose the right internal thoracic artery (RITA) to the RCA in a male taxi driver in a 17-second procedure. The recently dis-covered coronary angiogram was used on postopera-tive day 14 and demonstrated a patent graft. Ulti-mately, when this patient died 13 months later, the autopsy revealed a still-patent graft. This, however, was the only CABG procedure performed on a hu-man patient by Goetz and his team. In 1962, David Sabiston completed the first direct hand-sewn coro-nary anastomosis at Johns Hopkins Hospital, Balti-more, MD, United States. This was done as an off-pump, end-to-end anastomosis of a saphenous vein graft (SVG) to the RCA.^12^ When this patient died 3 days postoperatively from a stroke, Sabiston did not attempt another anastomosis for almost a decade.

The history of CABG is also full of achievements that were dictated by operative necessity. On No-vember 23, 1964, at Methodist Hospital, Houston, TX, US, Garrett, Dennis, and DeBakey scheduled a routine endarterectomy with patch grafting without cardiopulmonary bypass on a 42-year-old man using an SVG.^13^ In this case, however, the native vessel proved unsuitable because its lesion involved the left main artery bifurcation such that the team bypassed the LAD with an autologous SVG segment using end-to-side distal anastomosis. Angiography per-formed 7 years later revealed a patent vein graft and occluded native vessel. The team did not report the historic operation until 1973.

Visionary, daring surgeons continually contrib-uted to the evolution of CABG. Vasili Kolesov, from Russia, was considered the father of off-pump CABG, completing an off-pump RITA-to-RCA anastomosis on February 25, 1964.^14^ The RITA pedicle (Kolesov’s pedicle) was dissected, and the anastomosis per-formed on the RCA with atraumatic needles using 6-0 silk on the beating heart. The patient survived and reported no angina for 3 years. Notably, Kolesov was the first, and at that time the only, person in the world performing off-pump anastomoses. He was so ahead of his time that when he reported the out-comes of this and 11 other pioneering bypass sur-geries in 1967, at a conference in Leningrad, USSR, the plenum subsequently voted to accept a resolu-tion that surgical treatment of CAD had no future. Following that, in 1968, George Green in New York, US, performed the first LITA-to-LAD anastomosis, which is the cornerstone of modern-day CABG.^3^

### Standardization and Popularization: 1968–1970

Following the success of heart surgeries in the early 1960s, René Gerónimo Favaloro streamlined and popularized the operative technique, and an era involving a higher volume of surgical revascularization for treating ischemic heart disease unfolded.^15^

Favaloro was a brilliant medical student born in La Plata, Argentina, in 1923. His grandparents immi-grated from the Aeolian Islands off Sicily; his father was a carpenter and his mother a seamstress. He spent the first years of his career working as a pri-mary care physician in Jacinto Aráuz, a rural area of the La Pampa province, where his many other duties included performing general surgery. After 12 years of rural medicine and surgery, he was sent by his mentor to Cleveland Clinic, where he built his reputa-tion as the “father of coronary artery bypass surgery.”

Favaloro was the first cardiac surgeon to use a free SVG interposed end-to-end to the two transect-ed ends of the RCA after a lesion was excised, and the first to use the saphenous vein (SV) as the auto-logous vein conduit of choice. Autologous SVG seg-ments were used as bypass grafts, initially to the RCA and employing an end-to-end distal anasto-mosis, with the proximal end of the tied-off RCA.

Donald B. Effler served as Chief of Thoracic and Cardiovascular Surgery at Cleveland Clinic during those years (1949–1975) and, working with Sones, was a strong believer in the Vineberg procedure. He had devoted this work to the surgical treatment of myocardial ischemia. Of note, neither Favaloro nor Effler originally planned to use aortocoronary vein bypass, but quickly found that vein interpositions required two anastomoses and were of no use for ostial or very proximal lesions. It was for such lesions that they eschewed vein interposition and modified it into the bypass technique. The native vessel was still transected, and the distal anastomo-sis was still made end-to-end with the distal RCA; however, instead of making the proximal anastomo-sis with the other cut end of the native vessel, they moved more proximally by creating an anastomosis with the ascending aorta. The cut end of the proxi-mal coronary artery was then ligated. Favaloro then settled on performing vein bypass grafting with an end-to-side distal anastomosis, which quickly be-came the standard operation throughout the world.

Favaloro and his team often combined SVG of the RCA with single or double internal thoracic artery (ITA) myocardial implantation as a Vineberg procedure. Later he began to use single or double ITA grafting alone or in combination with vein by-pass. Indeed, from 1966 to the end of 1968, over 120 combined simultaneous revascularization proce-dures were performed at Cleveland Clinic, with a hospital mortality of 5%—equivalent to that of single revascularization procedures.^16^ At first, Favaloro and Effler often grafted the RCA on a beating heart, but they and others later routinely used cardiopul-monary bypass, especially when they then ventured into bypassing the left coronary artery (LCA) sys-tem. Favaloro became the first to perform vein bypass in patients in the setting of unstable angina or acute infarction, and the first to combine vein bypass with ITA implantation, valve replacement, or aneurysmectomy.

Though Favaloro was not the first to use vein bypass grafting in human subjects, it was the broad clinical application of his technique that revolution-ized the treatment of ischemic heart disease.^17^ He was meticulous in his review of studies and angio-grams from cases, prolific in publishing his analyses, and a dedicated teacher.^17^ Favaloro and Effler were initially treated with skepticism by the scientific community, but their rigorous scientific approach to the growing subject of myocardial revascularization allowed them to publish extensively.^15^ Favaloro was the first to write of teamwork in the field of cardiac surgery, thus laying the foundation for modern-day heart teams.

### Conduits and Techniques: 1970–Present

#### Conduits

The 1970s brought numerous innovations regarding conduit use. Favaloro reported the first CABG using SVG as a conduit in 1968, which remained the most used conduit for the first decades of the procedure. Since SVG disease due to intimal hyperplasia and accelerated atherosclerosis arose as a known complication of this conduit, other conduit options were developed.

In 1973, Carpentier was the first to use a radial artery conduit, and, in 1992, Acar revived its use with improvements.^18^,^19^ The radial artery has gained pop-ularity today due to its length (allowing it to reach distal coronary branches), thickness (allowing for ease of multiple anastomoses), diameter (similar to coronary arteries and not prone to kink easily), and ease of harvest.^20^ For these reasons, many surgeons consider the radial artery to be the second-best al-ternative to the RITA. Notable disadvantages include the possibility of spasm due to the thick tunica media, the potential for calcifications (Mönckeberg’s sclero-sis), intimal hyperplasia, and unequal caliber be-tween the proximal and distal ends.^21^ Although infrequent, neurologic hand complications relating to radial artery harvests have been reported.^22^

The 2021 American Association of Cardiology/ American Heart Association/Society of Cardiovascu-lar Angiography and Intervention (AAC/AHA/SCAI) Guideline for Coronary Artery Revascularization rec-ommended use of the radial artery in preference to the SV conduit for grafting of the second most im-portant non-LAD vessel in order to improve long-term cardiac outcomes.^23^ Indeed, in 2019, the RADIAL study showed a benefit to using the radial artery over the SV. Prevalence of myocardial infarc-tion, repeat revascularization, and mortality were all lower with radial artery use.^24^ The data, however, are not conclusive in that regard,^25^ although the largest trial comparing the radial artery to the SV did note a survival difference at 18 years of follow-up.^26^ In addition, some encouraging data are emerg-ing with regard to the improved patency of no-touch SVGs^27^ following the emergence of important data showing equivalent major adverse cardiovascular events (MACE) associated with open versus endo-scopic SVG harvesting.^28^

The ITA as a pedicle was initially used as part of the Vineberg procedure in the 1960s. Barner was first to use the ITA graft as a free graft in 1973.^29^ De-finitive clinical evidence supporting ITA use appeared in the mid-1980s, when Floyd Loop and Cleveland Clinic reported 10-year outcomes of ITA conduits versus total vein grafting.^30^ This showed that ITA use was associated with improved survival and re-duced risk of myocardial infarction, hospitalization, and need for repeat revascularization. These im-proved clinical outcomes correlated with improved patency of ITAs over SVGs. Studies have since re-vealed that the superiority of ITAs to SVGs has a physiologic basis in resistance to development of atherosclerosis and nitrous oxide production, bene-fiting the entire coronary system.^31^

Cleveland Clinic popularized, in the late 1990s, the use of bilateral ITA (BITA) grafting. Lytle showed that CABG using BITA was associated with greater survival and reduced need for reoperation as com-pared to single ITA grafting.^32^ Divergence of the sur-vival curves initially reported at 10-year follow-up was shown to continue in subsequent studies with 20 years of mean follow-up.^33^ This association of BITA superiority persisted regardless of whether the ITA was taken down as a pedicle or skeletonized graft, and despite diabetes status or sex of patients.^33^ Furthermore, following BITA grafting, the left and right ITAs seemed to have similar long-term patency and survival benefits regardless of configurations, although ITAs grafted to less-stenosed RCAs may have decreased patency.

In the context of these known long-term survival benefits of BITA grafting, it has been shown that RITA patency is affected more by target choice than by conduit configuration.^34^ A comparison of RITA occlusions between different RITA inflow configura-tions, by Bakaeen et al., showed high patency ir-respective of the inflow configuration. This estab-lishes that the priority should be whatever configu-ration optimizes the reach to important coronary targets, including the LAD. Earlier work by these investigators defined important coronary target ves-sels to be targeted by ITAs and demonstrated that, in BITA grafting, maximizing myocardium supplied by the ITAs improves long-term survival.^35^

Of note, the Arterial Revascularization Trial (ART), reported in 2019, revealed no survival or MACE difference at 10 years between single ITA and BITA grafting in multivessel CABG.^36^ However, the trial was criticized for high crossover from BITA to a single ITA arm and enrollment by inexperienced surgeons. Interestingly, in the as-treated analysis of ART, multiple arterial grafting (MAG) was associ-ated with improved outcomes, including survival consistent with a wealth of observational data. The ROMA trial aims to determine the effect of single versus multiple arterial revascularization (ITAs and radial arteries) for patients undergoing CABG.^37^

All in all, while ITA-to-LAD grafting has been the cornerstone of CABG since the late 1980s, MAG using at least two arterial grafts could improve sur-vival and decrease MACE in selected patients. This is why the Society of Thoracic Surgeons (STS) Clini-cal Practice Guidelines on arterial conduits for CABG encourage MAG and recommend supplementing the ITA-to-LAD graft with either a second ITA or radial artery in selected patients with multivessel CAD.^38^

The gastroepiploic artery (GEA) is another arterial conduit choice. In 1973, Edwards was the first to use it as a bypass for the RCA.^20^ In 1984, Pym and Suma were the first to report using the GEA for revascu-larization of the posterior marginal artery and RCA.^20^ This conduit is still used today, mainly for revascularizing the posterior descending artery or, occasionally, the distal segment of the RCA. (It is most commonly used in Asia.) Of course, there is an absolute contraindication for use of the right GEA if the patient had prior partial or complete gastrecto-my. The GEA is vulnerable to spasm and occlusion, but recent reports from experienced centers have demonstrated excellent results. Although less com-monly used today, use of the inferior epigastric artery was first described by Puig in 1990.^39^

The increased technical complexity and operative time as well as a longer latency period to realization of improved survival may all contribute to barriers to widespread adoption of MAG.^40^ To this day, more than 80% of conduits used in the United States are SV grafts.^38^ In a review of the STS database, MAG cases were 10.6% of all isolated CABG procedures between 2004 and 2015, with a significant decrease from 16% in 2004 to 9% in 2015. The most common revascularization strategy was radial artery multi-arterial graft (RA-MAG) followed by BITA-MAG.^41^

#### Techniques

The 1990s to 2000s was an era of new and innova-tive techniques drawing on the established concepts described. Both Buffolo and Benetti reported their relatively large, respective series of off-pump CABG with excellent outcomes.^42^,^43^ While off-pump CABG gained popularity in the decades that followed, sev-eral large, randomized, controlled trials and meta-analyses have failed to demonstrate clear outcome advantages.^44^ The results of the MASS-III trial, com-paring on-pump and off-pump CABG, were reported in 2010 and showed no difference in mortality, myo-cardial infarction, need for further revascularization, or stroke after 5 years.^45^ Three large multicenter trials that followed—ROOBY, CORONARY, and GOCABE—confirmed similar outcomes between the on- and off-pump approaches with regard to mortal-ity and major morbidity.^45^ Thus, use of off-pump CABG has declined. According to the STS, the per-centage of off-pump CABG procedures performed in 2002 was 23%, 17% in 2012, and 12% in 2021.^46^ For now, indications are that off-pump CABG can be beneficial in high-risk subgroups, but surgical ex-perience, skill, and preference are important factors for outcomes in considering on- versus off-pump ap-proaches, as these can be associated with dimin-ished long-term survival when not done by surgeons with expert-level skills in this area.^47^

Other innovations arose in the 1990s. In 1996, Calafiore published work on minimally invasive di-rect coronary artery bypass of the LITA to LAD, and Angelini did important work on hybrid revascular-ization.^48^ The boundaries of minimally invasive CABG were shifted in 1998 when Loulmet published important work on robotically assisted totally endoscopic coronary artery bypass (TECAB).^49^ In 2000, Falk and Mohr introduced further important work in the area of robotically assisted TECAB.^50^

There are two different techniques: (1) TECAB, where the ITA is harvested and the anastomosis done from the console through the thoracic ports with or without arrested heart, and (2) hybrid CABG, consisting of robotic, minimally invasive harvesting of the LITA, which is then used in hybrid coronary revascularization with a direct hand-sewn LITA-to-LAD anastomosis through a mini-thoracotomy. The latter procedure is more commonly performed today.

Adoption of these procedures thus far has been limited to very few dedicated centers.^51^ Even today, robotic surgery accounts for less than 1% of CABG procedures in the United States.^52^ This is, at least in part, due to relative lack of data supporting the ben-efits from these procedures, somewhat higher costs, longer operative times, need for specific training, and difficulty in teaching robotic multiarterial CABG and achieving complete revascularization reliably.^53^ A steep learning curve has been noted: in more than 1,000 robotically-assisted CABG procedures, an in-flection point is seen at a surgeon’s tenth procedure with respect to a composite measure of conversion, reoperation, major morbidity or mortality, and over-all success.^54^

Ruel and McGinn made important contributions to the evolution of minimally invasive CABG without the use of robotic or endoscopic adjuncts (referred to as MICS CABG).^55^ The ongoing MIST trial will certainly provide guidance based on observed out-comes.^56^

Finally, the development of intraoperative quality control methods has also contributed to the evolu-tion of streamlined techniques and improved out-comes. One example is transit time flow measure-ment (TTFM), which allows for intraoperative evalu-ation of coronary graft flow wherein an ultrasound probe facilitates measurement of blood flow volume through the graft. A systematic review yielded an expert consensus that use of TTFM has a favorable cost-benefit ratio based on evidence supporting an association between TTFM readings of graft patency and postoperative clinical outcomes.^57^

## CABG VERSUS PCI

In 1977, Gruntzig performed the first percutaneous coronary intervention (PCI) to dilate a LAD stenosis using balloon angioplasty.^58^ Within a decade, bare-metal stents were being inserted percutaneously into coronary arteries to prevent in-stent restenosis and recoil, and, shortly thereafter, drug-eluting stents were developed and improved to prevent stent thrombosis and restenosis.^59^

Both PCI and CABG provide symptomatic relief for patients, but repeat procedures are required more frequently after PCI than after CABG.^60^ The CABG procedure remains the gold standard for sur-gery in patients with multivessel CAD and is pre-ferred for anatomically high-risk patients with left main disease, diabetes, or ventricular dysfunction.^3^ It provides the best option for complete and durable revascularization and is the only revascularization procedure to offer a survival advantage over medical therapy in stable CAD.^61^

The 2021 AAC/AHA/SCAI Guideline for Coro-nary Artery Revascularization outlines scenarios where CABG may be preferred to PCI and, at the same time, emphasizes the importance of a heart team approach to decision-making.^23^ Some major trials and work in this area include SYNTAX,^62^ PRE-COMBAT,^63^ STITCH,^64^ ASCERT,^65^ FREEDOM,^66^ BEST,^67^ EXCEL,^68^ NOBLE,^69^ and FAME III^70^ (Table 1). It is important to note that these trials were limited to patients with lower-complexity CAD and those who were fit enough for equipoise between CABG and PCI, and long-term follow-up is sparse.

**Table 1:** Outcomes of CABG Versus PCI for Coronary Revascularization.

Year	Study	Result	Notes
2009	SYNTAX^62^	Lower MACCE with CABG rates in patients with severe coronary disease as defined by SYNTAX score	Supports CABG as the standard of care for patients with 3-vessel disease or complex left main CAD
2011	PRECOMBAT^63^	PCI with sirolimus-eluting stents was non-inferior to CABG in patients with unprotected left main CAD with respect to MACCE at 1 year	Both groups had similar rates of death/MI/stroke. Occurrence of ischemia-driven, target-vessel revascularization at 2 years was lower in CABG than PCI
2011	STITCH^64^	Compared conservative treatment with medical therapy vs. medical therapy + CABG in patients with CAD and LV dysfunction. No significant differences in all-cause mortality, though rate of cardiac-cause hospitalization was lower with CABG	10-year follow-up, reported in 2019, concluded that CABG reduces all-cause mortality, and cardiovascular and heart failure hospitalizations^71^
2012	ASCERT^65^	No significant difference in mortality between patients ≥65 years with 2- or 3-vessel disease undergoing CABG vs. PCI at 1 year. Lower mortality with CABG than PCI at 4-year follow-up	
2012	FREEDOM^66^	CABG superior to PCI in patients with diabetes and multivessel disease; signifi-cantly reduced death and MI at 5 years	Some increased risk of strokes in the CABG group
2015	BEST^67^	For multivessel CAD patients, higher MACE in PCI vs. CABG group; higher spontaneous MI and repeat revasculariza-tion after PCI vs. CABG	Similar comparison to FREEDOM trial
2019	EXCEL^68^	At 5 years, no significant difference in death/stroke/MI between PCI and CABG patients with left main CAD of low or intermediate anatomical complexity	Some methodological controversy:^72^ incidence of all-cause mortality significantly higher in PCI group at 5 years, but all-cause mortality classified as a secondary not primary endpoint. Repeat revascu-larization was significantly higher in PCI vs. CABG groups (also not a primary endpoint). Occurrence of composite death, stroke, MI score shifted from favoring PCI to favoring CABG after 30-days’ follow-up, which may indicate CABG is preferred in patients with a higher life expectancy
2020	NOBLE^69^	PCI had inferior 5-year clinical outcomes in patients with left main disease as compared with CABG	Both procedures had similar rates of mortality, but PCI had higher rates of repeat revascularization and of non-procedural MI
2022	FAME III^70^	Examined whether FFR-guided PCI was non-inferior to CABG in 1-year composite outcome (death, MI, stroke, or repeat revascularization) in patients with 3-vessel disease. The study did not show non-inferiority, and CABG resulted in lower incidence of composite outcome	30-day CABG mortality was 0.3%, identical to that of PCI
2023	Meta-analysis of Randomized Trials^73^	During 5-year follow-up, PCI showed higher incidence of all-cause mortality, MI, and repeat coronary revascularization	Meta-analysis comparing CABG and PCI for treatment of left main or multivessel disease Elevated risk of stroke in 30-day post-operative period for CABG, but no long-term difference at 5-year follow-up

CABG, coronary artery bypass graft; CAD, coronary artery disease; FFR, fractional flow reserve; LV, left ventricle/ventricular; MACCE, major adverse cardiovascular and cerebrovascular events; MACE, major adverse cardiovascular events; MI, myocardial infarction; PCI, percutaneous coronary intervention.

Regardless of the revascularization procedure used, the importance of guideline-directed medical therapy (GDMT) cannot be overstated. Studies have shown that compliance with GDMT in contemporary coronary revascularization trials is significantly lower after CABG than after PCI.^74^ The point has been made that this should affect how we draw compari-sons between these study groups.^61^ The case has also been made that among patients who are non-adherent to GDMT, CABG affords a better major adverse cardiovascular event (MACE)-free survival, and, as a result, the likelihood of patient compliance may be useful in informing shared clinical decision-making.^75^

Today, hybrid techniques are also being devel-oped. Given the significant benefits of LITA-to-LAD anastomosis, some centers have developed hybrid coronary revascularization, wherein cardiac surgeons and interventional cardiologists work together to per-form (1) LITA-LAD bypass, typically with a mini-mally invasive procedure, and (2) PCI on non-LAD arteries, both scheduled and performed within a pre-defined time interval in patients with multivessel dis-ease (as opposed to use of SVGs for these other tar-gets).^76^ The benefit is a limited-access, sternotomy-sparing approach. This method is still new, with roughly 2,000 patients making up the total of hybrid coronary revascularization studies and trials.^52^ Early work suggests possible benefits with respect to occurrence of stroke, infection rate, and recovery time, but observation in this area is ongoing.^77^

## CURRENT CABG TRENDS

The STS Adult Cardiac Surgery database (ACSD) provides a useful perspective on trends in the field. According to the 2022 ACSD update, isolated CABG continues to represent more than 70% of all cardiac surgery cases.^78^ Roughly 400,000 CABG procedures are performed in the United States annually, for an estimated total expenditure of $16 billion, compared with roughly 950,000 PCIs performed annually, for an estimated total expenditure of $12 billion.^79^

**Figure 2:**
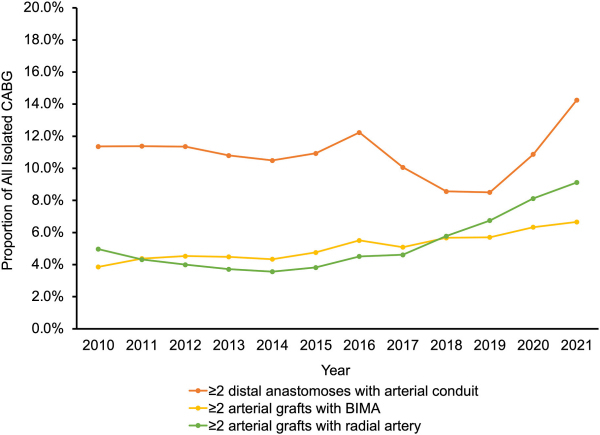
Use of MAG in Isolated CABG: Two or More Distal Anastomoses with Arterial Conduit (Red Line); Two or More Arterial Grafts with BITA (Yellow Line); and Two or More Arterial Grafts with Right Atrium (Green Line).^78^ BIMA, bilateral internal mammary artery; BITA, bilateral internal thoracic artery; CABG, coronary artery bypass graft; MAG, multiple arterial grafting. Reprinted from Kim et al.,^78^ copyright 2023, with permission from Elsevier.

Overall, in the United States, MAG remains some-what underused and limited to selected centers, with only 14% and 21% of institutions performing more than 30 BITA and radial artery multiarterial cases, respectively, in 2018 to 2019 (Figure 2).^79^

**Figure 3:**
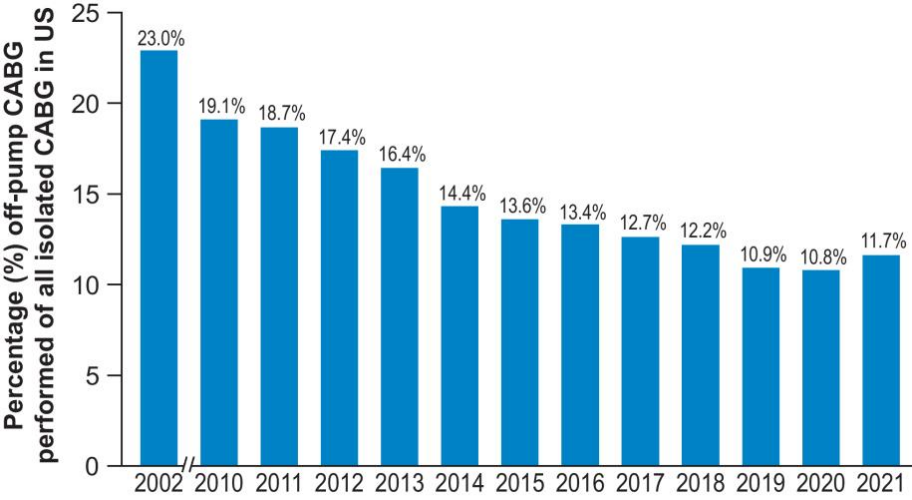
Performance of Off-pump CABG over Time CABG, coronary artery bypass graft.

With regard to the use of cardiopulmonary by-pass, there has been a significant decline in the num-ber of off-pump CABG operations for the reasons noted earlier (from 23% of cases in 2002, to 17% in 2012,^80^ to just under 12% in 2021^78^) (Figure 3).

With respect to robotically assisted CABG, its adoption has been limited. It was used in 0.97% of all CABG procedures in the United States from 2006 to 2012.^52^

According to a recent examination of the STS database, reoperative CABG represents roughly 4.83% of all isolated CABG procedures nationally. Redo CABG has declined over time, from 6.0% in 2000 to 3.4% in 2009.^81^ Patients undergoing redo are typically older, have decreased left ventricular function, and frequently have more advanced comor-bidities and a higher atherosclerotic burden than those undergoing primary CABG.^82^ The preferred therapeutic option is typically PCI, but in some circumstances reoperative CABG is required.^23^,^83^ For instance, redo CABG remains appropriate for many patients who cannot undergo PCI or would have a survival benefit from arterial conduit grafting to the LAD. This would be the case in new, significant LAD stenosis, and also for early graft failure or later presentations after primary CABG with the same indications as for primary CABG.^84^ Careful patient selection, appropriate surgical experience, and peri-operative strategies remain essential for this complex operation.^85^

## THE FUTURE

Drawing on the historical evolution of CABG and current trends, we can look toward many future areas of improvement and innovation.

One important area of new techniques involves surgical revascularization in patients with ischemic cardiomyopathy and heart failure. In 2021, an American Association for Thoracic Surgery Expert Consensus Group examined the existing evidence and guidelines to provide clinical practice insights for patients with CAD complicated by ischemic cardiomyopathy and heart failure.^86^ This Group sets forth a framework approach that involves certain revascularization modalities for specific populations of heart failure patients as part of a broad heart team approach including, where appropriate, me-chanical cardiac support (MCS). As this guidance document notes, high-level evidence in this domain is sparse and remains an area in need of future focus. It is clear that MCS does and will continue to play an important role in managing patients in this population. Future work is likely to further fine-tune perioperative roles of MCS devices, including RV support, in this higher-risk population.

With respect to conduits, more definitive answers on MAG and optimal grafting will evolve in the fu-ture. This notwithstanding, surgeons with superior outcomes from MAG are likely to continue using it.

Another area of future work may involve expan-sion of intraoperative quality control measures. As noted above, TTFM assessment of patency intraop-eratively—particularly for arterial grafts and grafts to the LAD—guides the need for graft revisions and can optimize patency and outcomes.^57^ Similarly, the principles of Enhanced Recovery After Surgery (ERAS) are likely to be increasingly applied to car-diac surgery; ERAS is a multimodal, perioperative care pathway to achieve early recovery after surgery. The application of ERAS principles broadly has been associated with a 30% to 40% reduction in the num-ber of complications in several different operations and with shortened recovery times.^87^

The role of biomarkers in perioperative planning and prognostication is an area ripe for added focus as well. Markers of inflammation, vascular dysfunc-tion, myocardial remodeling, and oxidative stress have shown promise in augmenting typical markers, like troponins and natriuretic peptides, for preoper-ative optimization and postoperative surveillance.^88^ Of note, it has been theorized that arterial grafts can protect the downstream coronary bed from progres-sion of atherosclerosis through production of anti-inflammatory and antithrombotic mediators.^89^ In this way, future studies focused on the cellular-level bases for certain risk factors and pathologic out-comes will be helpful.

Another important area of future focus broadly includes all new, innovative approaches to improv-ing outcomes. The STS 2021 update on outcomes, quality, and research highlights important trends in quality metrics.^46^ It features a study analyzing pa-tients who underwent isolated CABG between 2011 and 2018 where a composite socioeconomic metric (the Distressed Communities Index) was significant-ly associated with mortality and the composite of morbidity and mortality.^90^ This indicates that pa-tients from certain socioeconomic backgrounds may be at higher risk for adverse events and death after CABG and highlights the importance of identifying high-risk patients. Similarly, the 2022 STS ACSD update also noted sex differences in revasculariza-tion techniques among CABG patients—specifically that female patients were significantly less likely to receive guideline-concordant revascularization, in-cluding LITA-to-LAD grafting, MAG, and complete revascularization.^78^ Future work targeting improved outcomes for at-risk populations will be an im-portant area of focus.

Machine-learning algorithms have been increas-ingly recognized as a possible method for predicting mortality and morbidity. In one recent study of al-most 400,000 isolated CABG procedures, it was shown that machine-learning models that amass and analyze preoperative and intraoperative vari-ables demonstrated improved prediction over either set of variables alone.^91^ The era of artificial intelli-gence, big data, wearable medical devices, digital health, and personalized medicine is already here and is bound to revolutionize cardiac care.^92^

Finally, there is some movement toward pro-grammatic and surgeon specialization in CABG, which in itself has shown association with improved outcomes. One institution implemented a subspe-cialized coronary surgery program and examined outcomes before and after its use.^93^ This study found that CABGs done in the specialization period had shorter bypass and clamp times, increased use of BITA grafting, fewer complications, and reduced overall operative mortality. Within this context, it has been noted that recognition of CABG as a sub-specialty could bring with it dedicated training programs.^94^ So many of the studies and trials described in this paper conclude that surgeon experience can be a critical component of improved outcomes, especially in the context of novel areas of CABG. Therefore, there is important future work to be done on what CABG subspecialization may look like with respect to education, training, and certification.

## CONCLUSION

The CABG procedure is the most commonly per-formed major cardiac operation and the most stud-ied intervention worldwide. With well over 20,000 cited articles on PubMed, there is no other interven-tion in adult cardiac surgery that has been more extensively studied.

Since the first CABG procedure was completed in 1960, many bold individual surgeons and teams contributed to the success that it is today. The land-scape of CABG has changed through the years. It has become less common, but more challenging in the context of older and sicker patients with extensive atherosclerotic burden. Improved techniques and standardization through the work of pioneers like Favaloro have broadened its accessibility and suc-cess; the introduction and spread of PCI and sub-sequent trials examining both approaches have streamlined its indication and confirmed its efficacy. New innovations using minimally invasive tech-niques, off-pump options, and robotic assistance have allowed for the possibility of tailoring tech-niques to specific patient populations.

With respect to quality improvement, CABG has been the first and only operation in adult cardiac surgery with registries owned at the state level in the United States and internationally. Today LITA-to-LAD grafting is a mandated quality marker in CABG and tracked extensively. Intraoperative graft patency assessment is gaining a foothold and is a critical adjunct for complex multiarterial grafting and mini-mally invasive techniques.

The CABG operation developed out of a sequence of historical events: a story of failures, disappoint-ments, fortuitous discoveries, successes, and opera-tional accidents in many operating rooms all over the world. It is a story of innovators, visionaries, and pioneers who contributed with perseverance to the evolution of one of the most advanced surgical interventions in history. But, more than that, the story of CABG also proves that only a multidisci-plinary and collaborative approach to medicine can achieve exceptional results. As Dr Favaloro said in 1997, “Medicine depends on evolution … by means of the work of many contributors. I could claim many ‘firsts.’ I never did, because to me ‘we’ is more important than ‘I.’”^95^ It is from this foundation of collaboration and innovation that CABG will contin-ue to evolve.

## Abbreviations

AAC/AHA/SCAI: American Association of Cardiology/American Heart Association/Society of Cardiovascular Angiography and Intervention
ACSD: adult cardiac surgery database
BITA: bilateral internal thoracic artery
CABG: coronary artery bypass graft
CAD: coronary artery disease
ERAS: enhanced recovery after surgery
GDMT: guideline-directed medical therapy
GEA: gastroepiploic artery
ITA: internal thoracic artery
LAD: left anterior descending coronary artery
LITA: left internal thoracic artery
MACE: major adverse cardiovascular events
MAG: multiple arterial grafting
PCI: percutaneous coronary intervention
RA: right atrium
RCA: right coronary artery
RITA: right internal thoracic artery
STS: Society of Thoracic Surgeons
SVG: saphenous vein graft
TECAB: totally endoscopic coronary artery bypass
TTFM: transit time flow measurement.

## References

[1] Head SJ, Milojevic M, Taggart DP, Puskas JD (2017). Current practice of state-of-the-art surgical coronary revascularization. Circulation.

[2] Bachar BJ, Manna B (2023). Coronary Artery Bypass Graft. StatPearls [Internet].

[3] Melly L, Torregrossa G, Lee T, Jansens JL, Puskas JD (2018). Fifty years of coronary artery bypass grafting. J Thorac Dis.

[4] Sandler G, Slesser BV, Lawson CW (1967). The Beck operation in the treatment of angina pectoris. Thorax.

[5] Katrapati P, George JC (2008). Vineberg operation: a review of the birth and impact of this surgical technique. Ann Thorac Surg.

[6] Rozsival V (2006). Outcome of Vineberg's operation after 31 years. Heart.

[7] Nakayama DK (2018). The development of extracorporeal membrane oxygenation. Am Surg.

[8] Cheng TO (2003). First selective coronary arteriogram. Circulation.

[9] Proudfit WL (1986). In memoriam F. Mason Sones, Jr., M.D. 1918–1985 the man and his work. Cleve Clin Q.

[10] Ryan TJ (2002). The coronary angiogram and its seminal contributions to cardiovascular medicine over five decades. Trans Am Clin Climatol Assoc.

[11] Blondeau P, Dubost C (1962). Ann Chir Thorac Cardiovasc.

[12] Sabiston DC (1974). The William F. Rienhoff Jr lecture. The coronary circulation. Johns Hopkins Med J.

[13] Garrett HE, Dennis EW, DeBakey ME (1973). Aortocoronary bypass with saphenous vein graft. Seven-year follow-up. JAMA.

[14] Olearchyk AS, Vasilii I (1988). Kolesov. A pioneer of coronary revascularization by internal mammary-coronary artery grafting. J Thorac Cardiovasc Surg.

[15] Favaloro RG (1969). Saphenous vein graft in the surgical treatment of coronary artery disease. Operative technique. J Thorac Cardiovasc Surg.

[16] Favaloro RG, Effler DB, Groves LK, Razavi M, Lieberman Y (1969). Combined simultaneous procedures in the surgical treatment of coronary artery disease. Ann Thorac Surg.

[17] Bakaeen FG, Blackstone EH, Pettersson GB, Gillinov AM, Svensson LG (2018). The father of coronary artery bypass grafting: René Favaloro and the 50th anniversary of coronary artery bypass grafting. J Thorac Cardiovasc Surg.

[18] Carpentier A, Guermonprez JL, Deloche A, Frechette C, DuBost C (1973). The aorta-to-coronary radial artery bypass graft: a technique avoiding pathological changes in grafts. Ann Thorac Surg.

[19] Acar C, Jebara VA, Portoghese M (1992). Revival of the radial artery for coronary artery bypass grafting. Ann Thorac Surg.

[20] Martínez-González B, Reyes-Hernández CG, Quiroga-Garza A (2017). Conduits used in coronary artery bypass grafting: a review of morphological studies. Ann Thorac Cardiovasc Surg.

[21] Shanahan CM, Cary NRB, Salisbury JR, Proudfoot D, Weissberg PL, Edmonds ME (1999). Medial localization of mineralization-regulating proteins in association with Mönckeberg's sclerosis: evidence for smooth muscle cell-mediated vascular calcification. Circulation.

[22] Moon MR, Barner HB, Bailey MS (2004). Long-term neurologic hand complications after radial artery harvesting using conventional cold and harmonic scalpel techniques. Ann Thorac Surg.

[23] Lawton JS, Tamis-Holland JE, Bangalore S, Writing Committee Members (2022). 2021 ACC/AHA/SCAI Guideline for Coronary Artery Revascularization: A report of the American College of Cardiology/American Heart Association Joint Committee on Clinical Practice Guidelines. J Am Coll Cardiol.

[24] Gaudino M, Bakaeen FG, Benedetto U (2019). Arterial grafts for coronary bypass: a critical review after the publication of ART and RADIAL. Circulation.

[25] Nissen SE, Bakaeen FG (2020). Coronary revascularization strategies: making sense of sparse, limited-quality data. JAMA.

[26] Goldman S, McCarren M, Sethi GK (2022). Long-term mortality follow-up of radial artery versus saphenous vein in coronary artery bypass grafting: a multicenter, randomized trial. Circulation.

[27] Gomes WJ, Kim KB, Pinheiro BB, Souza DSR (2022). The no-touch saphenous vein graft in coronary artery bypass surgery. Towards a new standard?. Braz J Cardiovasc Surg.

[28] Zenati MA, Bhatt DL, Bakaeen FG (2019). Randomized trial of endoscopic or open vein-graft harvesting for coronary-artery bypass. N Engl J Med.

[29] Barner HB (1973). The internal mammary artery as a free graft. J Thorac Cardiovasc Surg.

[30] Loop FD, Lytle BW, Cosgrove DM (1986). Influence of the internal-mammary-artery graft on 10-year survival and other cardiac events. N Engl J Med.

[31] Kraler S, Libby P, Evans PC (2021). Resilience of the internal mammary artery to atherogenesis: shifting from risk to resistance to address unmet needs. Arterioscler Thromb Vasc Biol.

[32] Lytle BW, Blackstone EH, Loop FD (1999). Two internal thoracic artery grafts are better than one. J Thorac Cardiovasc Surg.

[33] Lytle BW, Blackstone EH, Sabik JF, Houghtaling P, Loop FD, Cosgrove DM (2004). The effect of bilateral internal thoracic artery grafting on survival during 20 postoperative years. Ann Thorac Surg.

[34] Bakaeen FG, Ghandour H, Ravichandren K (2022). Right internal thoracic artery patency is affected more by target choice than conduit configuration. Ann Thorac Surg.

[35] Bakaeen FG, Ravichandren K, Blackstone EH (2020). Coronary artery target selection and survival after bilateral internal thoracic artery grafting. J Am Coll Cardiol.

[36] Taggart DP, Benedetto U, Gerry S (2019). Bilateral versus single internal-thoracic-artery grafts at 10 years. N Engl J Med.

[37] Joint Clinical Trials Office ROMA Trial: clinical trial details. Weill Cornell Medicine [website].

[38] Aldea GS, Bakaeen FG, Pal J (2016). The Society of Thoracic Surgeons Clinical Practice Guidelines on Arterial Conduits for Coronary Artery Bypass Grafting. Ann Thorac Surg.

[39] Puig LB, Ciongolli W, Cividanes GVL (1990). Inferior epigastric artery as a free graft for myocardial revascularization. J Thorac Cardiovasc Surg.

[40] Akhrass R, Bakaeen FG (2021). The 10 commandments for multiarterial grafting. Innovations (Phila).

[41] Schwann TA, Habib RH, Wallace A (2018). Operative outcomes of multiple-arterial versus single-arterial coronary bypass grafting. Ann Thorac Surg.

[42] Buffolo E, de Andrade CS, Branco JN, Teles CA, Aguiar LF, Gomes WJ (1996). Coronary artery bypass grafting without cardiopulmonary bypass. Ann Thorac Surg.

[43] Benetti FJ, Naselli G, Wood M, Geffner L (1991). Direct myocardial revascularization without extracorporeal circulation. Experience in 700 patients. Chest.

[44] Patel V, Unai S, Gaudino M, Bakaeen F (2019). Current readings on outcomes after off-pump coronary artery bypass grafting. Semin Thorac Cardiovasc Surg.

[45] Hueb W, Lopes NH, Pereira AC (2010). Five-year follow-up of a randomized comparison between off-pump and on-pump stable multivessel coronary artery bypass grafting. The MASS III Trial Circulation.

[46] Bowdish ME, D'Agostino RS, Thourani VH (2021). STS Adult Cardiac Surgery Database: 2021 update on outcomes, quality, and research. Ann Thorac Surg.

[47] Dimagli A, Weiss AJ, Bakaeen FG (2022). Off-pump coronary artery bypass grafting-not for every patient, not for every surgeon. JAMA Surg.

[48] Rivetti LA, Gandra SMA (1997). initial experience using an intraluminal shunt during revascularization of the beating heart. Ann Thorac Surg.

[49] Loulmet D, Carpentier A, d'Attellis N (1999). Endoscopic coronary artery bypass grafting with the aid of robotic assisted instruments. J Thorac Cardiovasc Surg.

[50] Falk V, Diegler A, Walther T, Autschbach R, Mohr FW (2000). Developments in robotic cardiac surgery. Curr Opin Cardiol.

[51] Weiss AJ, Frankel WC, Bakaeen FG (2021). Commentary: Beyond the horizon of evidence in robotic totally endoscopic coronary artery bypass grafting. JTCVS Tech.

[52] Gaudina M, Bakaeen F, Davierwala P (2018). New strategies for surgical myocardial revascularization. Circulation.

[53] Dimeling G, Bakaeen L, Khatri J, Bakaeen FG (2021). CABG: when, why, and how?. Cleve Clin J Med.

[54] Patrick WL, Iyengar A, Han JJ (2021). The learning curve of robotic coronary arterial bypass surgery: a report from the STS database. J Card Surg.

[55] McGinn JT, Usman S, Lapierre H, Pothula VR, Mesana TG, Ruel M (2009). Minimally invasive coronary artery bypass grafting: dual-center experience in 450 consecutive patients. Circulation.

[56] Calafiore AM, Angelini GD, Bergsland J, Salerno TA (1996). Minimally invasive coronary artery bypass grafting. Ann Thorac Surg.

[57] Gaudino M, Sandner S, Di Giammarco G (2021). The use of intraoperative transit time flow measurement for coronary artery bypass surgery: systematic review of the evidence and expert opinion statements. Circulation.

[58] Gruntzig A (1978). Transluminal dilatation of coronaryartery stenosis. Lancet.

[59] Simard T, Hibbert B, Ramirez FD, Froeschl M, Chen YX, O'Brien ER (2014). The evolution of coronary stents: a brief review. Can J Cardiol.

[60] Rihal CS, Raco DL, Gersh BJ, Yusuf S (2003). Indications for coronary artery bypass surgery and percutaneous coronary intervention in chronic stable angina: review of the evidence and methodological considerations. Circulation.

[61] Bakaeen FG, Chu D, Dayan V (2023). 2021 Coronary Revascularization Guidelines-lessons in the importance of data scrutiny and reappraisal of evidence. JAMA Surg.

[62] Serruys PW, Morice MC, Kappetein AP (2009). Percutaneous coronary intervention versus coronary-artery bypass grafting for severe coronary artery disease. N Engl J Med.

[63] Park SJ, Kim YH, Park DW (2011). Randomized trial of stents versus bypass surgery for left main coronary artery disease. N Engl J Med.

[64] Velazquez EJ, Lee KL, Deja MA (2011). Coronary-artery bypass surgery in patients with left ventricular dysfunction. N Engl J Med.

[65] Weintraub WS, Grau-Sepulveda MV, Weiss JM (2012). Comparative effectiveness of revascularization strategies. N Engl J Med.

[66] Farkouh ME, Domanski M, Sleeper LA (2012). Strategies for multivessel revascularization in patients with diabetes. N Engl J Med.

[67] Park SJ, Ahn JM, Kim YH (2015). Trial of everolimuseluting stents or bypass surgery for coronary disease. N Engl J Med.

[68] Stone GW, Kappetein AP, Sabik JF (2019). Five-year outcomes after PCI or CABG for left main coronary disease. N Engl J Med.

[69] Holm NR, Mäkikallio T, Lindsay MM (2020). Percutaneous coronary angioplasty versus coronary artery bypass grafting in the treatment of unprotected left main stenosis: updated 5-year outcomes from the randomised, non-inferiority NOBLE trial. Lancet.

[70] Fearon WF, Zimmermann FM, De Bruyne B (2022). Fractional flow reserve-guided PCI as compared with coronary bypass surgery. N Engl J Med.

[71] Howlett JG, Stebbins A, Petrie M (2019). CABG improves outcomes in patients with ischemic cardiomyopathy: 10-year follow-up of the STICH Trial. JACC Heart Fail.

[72] Jahangiri M, Mani K, Yates MT, Nowell J (2020). The EXCEL Trial: the surgeons' perspective. Eur Cardiol.

[73] Formica F, Gallingani A, Tuttolomondo D (2023). Longterm outcomes comparison between surgical and percutaneous coronary revascularization in patients with multivessel coronary disease or left main disease: a systematic review and study level meta-analysis of randomized trials. Curr Probl Cardiol.

[74] Pinho-Gomes AC, Azevedo L, Ahn JM (2018). Compliance with guideline-directed medical therapy in contemporary coronary revascularization trials. J Am Coll Cardiol.

[75] Kurlansky P, Herbert M, Prince S, Mack M (2016). Coronary artery bypass graft versus percutaneous coronary intervention: meds matter: impact of adherence to medical therapy on comparative outcomes. Circulation.

[76] Harskamp RE, Bonatti JO, Zhao DX (2014). Standardizing definitions for hybrid coronary revascularization. J Thorac Cardiovasc Surg.

[77] Puskas JD, Halkos ME, DeRose JJ (2016). Hybrid coronary revascularization for the treatment of multi-vessel coronary artery disease: a multicenter observational study. J Am Coll Cardiol.

[78] Kim KM, Arghami A, Habib R (2023). The Society of Thoracic Surgeons Adult Cardiac Surgery Database: 2022 update on outcomes and research. Ann Thorac Surg.

[79] Saadat S, Habib R, Engoren M (2023). Multiarterial coronary artery bypass grafting practice patterns in the United States: analysis of the Society of Thoracic Surgeons Adult Cardiac Surgery Database. Ann Thorac Surg.

[80] Bakaeen FG, Shroyer ALW, Gammie JS (2014). Trends in use of off-pump coronary artery bypass grafting: results from the Society of Thoracic Surgeons Adult Cardiac Surgery Database. J Thorac Cardiovasc Surg.

[81] Ghanta RK, Kaneko T, Gammie JS, Sheng S, Aranki SF (2013). Evolving trends of reoperative coronary artery bypass grafting: an analysis of the Society of Thoracic Surgeons Adult Cardiac Surgery Database. J Thorac Cardiovasc Surg.

[82] Elbadawi A, Hamed M, Elgendy IY (2020). Outcomes of reoperative coronary artery bypass graft surgery in the United States. J Am Heart Assoc.

[83] Locker C, Greiten LE, Bell MR (2019). Repeat coronary bypass surgery or percutaneous coronary intervention after previous surgical revascularization. Mayo Clin Proc.

[84] Sabik JF, Blackstone EH, Houghtaling PL, Walts PA, Lytle BW (2005). Is reoperation still a risk factor in coronary artery bypass surgery?. Ann Thorac Surg.

[85] Bakaeen FG, Akras Z, Svensson LG (2018). Redo coronary artery bypass grafting. Indian J Thorac Cardiovasc Surg.

[86] Bakaeen FG, Gaudino M, Whitman G (2021). 2021 The American Association for Thoracic Surgery Expert Consensus Document: coronary artery bypass grafting in patients with ischemic cardiomyopathy and heart failure. J Thorac Cardiovasc Surg.

[87] Ljungqvist O (2019). The enhanced recovery after surgery in cardiac surgery revolution. JAMA Surg.

[88] Yancy CW, Jessup M, Bozkurt B (2017). 2017 ACC/ AHA/HFSA Focused Update of the 2013 ACCF/AHA Guideline for the Management of Heart Failure: a report of the American College of Cardiology/American Heart Association Task Force on Clinical Practice Guidelines and the Heart Failure Society of America. J Am Coll Cardiol.

[89] Lüscher TF, Diederich D, Siebenmann R (1988). Difference between endothelium-dependent relaxation in arterial and in venous coronary bypass grafts. New Engl J Med.

[90] Mehaffey JH, Hawkins RB, Charles EJ (2020). Distressed communities are associated with worse outcomes after coronary artery bypass surgery. J Thorac Cardiovasc Surg.

[91] Mori M, Durant TJS, Huang C (2021). Toward dynamic risk prediction of outcomes after coronary artery bypass graft: improving risk prediction with intraoperative events using gradient boosting. Circ Cardiovasc Qual Outcomes.

[92] Vandenberk B, Chew DS, Prasana D, Gupta S, Exner DV (2023). Successes and challenges of artificial intelligence in cardiology. Front Digit Health.

[93] Watkins AC, Ghoreishi M, Maassel NL (2018). Programmatic and surgeon specialization improves mortality in isolated coronary bypass grafting. Ann Thorac Surg.

[94] Bakaeen FG, Johnston DR, Svensson LG (2021). Commentary: Coronary artery bypass grafting as a subspecialty: hype or reality. J Thorac Cardiovasc Surg.

[95] Konstantinov IE (2019). René Favaloro and the fatherhood of the coronary bypass operation: lest we forget. J Thorac Cardiovasc Surg.

